# Remodelled ribosomal populations synthesize a specific proteome in proliferating plant tissue during cold

**DOI:** 10.1098/rstb.2023.0384

**Published:** 2025-03-06

**Authors:** Federico Martinez-Seidel, Pipob Suwanchaikasem, Dione Gentry-Torfer, Yogeswari Rajarathinam, Alina Ebert, Alexander Erban, Alexandre Firmino, Shuai Nie, Michael Leeming, Nicholas Williamson, Ute Roessner, Joachim Kopka, Berin A. Boughton

**Affiliations:** ^1^ Molecular Physiology Department, Max Planck Institute of Molecular Plant Physiology, Potsdam-Golm, Germany; ^2^ Department of Biochemistry and Molecular Genetics, University of Colorado School of Medicine, Aurora, CO, USA; ^3^ RNA Bioscience Initiative, University of Colorado School of Medicine, Aurora, CO, USA; ^4^ School of BioSciences, The University of Melbourne, Parkville, Victoria, Australia; ^5^ Bio21 Institute of Molecular Science and Biotechnology, The University of Melbourne, Parkville, Victoria, Australia; ^6^ School of Chemistry, The University of Melbourne, Parkville, Victoria, Australia; ^7^ Department of Biochemistry and Molecular Biology, The University of Melbourne, Parkville, Victoria, Australia; ^8^ Research School of Biology, The Australian National University, Acton, Australia; ^9^ La Trobe Institute of Sustainable Agriculture and Food, La Trobe University, Bundoora, Victoria 3083, Australia

**Keywords:** ribosome remodelling, kinetic mass spectrometry, protein synthesis rates, ribosome heterogeneity, translation initiation, ribosome specialization

## Abstract

Plant acclimation occurs through system-wide mechanisms that include proteome shifts, some of which occur at the level of protein synthesis. All proteins are synthesized by ribosomes. Rather than being monolithic, transcript-to-protein translation machines, ribosomes can be selective and cause proteome shifts. In this study, we use apical root meristems of germinating seedlings of the monocotyledonous plant barley as a model to examine changes in protein abundance and synthesis during cold acclimation. We measured metabolic and physiological parameters that allowed us to compare protein synthesis in the cold to optimal rearing temperatures. We demonstrated that the synthesis and assembly of ribosomal proteins are independent processes in root proliferative tissue. We report the synthesis and accumulation of various macromolecular complexes and propose how ribosome compositional shifts may be associated with functional proteome changes that are part of successful cold acclimation. Our study indicates that translation initiation is limiting during cold acclimation while the ribosome population is remodelled. The distribution of the triggered ribosomal protein heterogeneity suggests that altered compositions may confer 60S subunits selective association capabilities towards translation initiation complexes. To what extent selective translation depends on heterogeneous ribo-proteome compositions in barley proliferative root tissue remains a yet unresolved question.

This article is part of the discussion meeting issue ‘Ribosome diversity and its impact on protein synthesis, development and disease’.

## 1. Introduction

Cold acclimation is a physiological challenge for sessile plant organisms [1–4]. In addition to extensively studied transcriptional responses [4–8], cold triggers translational reprogramming events that are thought to enhance acclimation [9–11]. Thus, translation may provide an additional regulatory layer during cold acclimation that integrates signals from cellular processes and shapes the proteome influencing gene expression, perhaps to a greater extent than a remodelled transcriptome [12–16]. We previously showed that after sensing cold, germinating barley seedlings halt growth [14], and yet, as compared to control plants, these seedlings continue to accumulate proteins functionally related to translation, particularly in proliferating tissues such as the root tips [14]. Irrespective of the mechanism, the centrality of protein translation for responses to cold seems evident, and the accumulation of structural ribosomal proteins (r-proteins) correlates with a cold-induced shift in the proteome. This shift includes the differential accumulation of protein factors related to ribosome assembly and translation initiation [14], suggesting that the accumulated proteome not only provides ribosomes with an autocatalysis-like function but also that translation initiation may contribute to the selection of messenger ribonucleic acid (mRNAs) that get translated after a cold stimulus.

Similar to barley, *Arabidopsis thaliana* (Arabidopsis) accumulates ribosomes after a cold shift, and the accumulated population of ribosomes exhibits altered r-protein compositions [13,15], suggesting that temperature shifts may trigger functional heterogeneity in the ribosomal population. Ribosome functional heterogeneity can contribute to proteome shifts via selective translation of the transcriptome and is referred to as ribosome specialization [17–19]. Specialization of ribosomes typically involves translational events that drive a response to external [20–23] or developmental [24–26] cues. For example, in the context of cold stress, the preferred translation of groups of proteins could facilitate acclimation. Remarkably, abundance changes of r-proteins in cold-acclimating barley seedlings [14] hold the potential for the assembly of heterogeneous ribosomes that may be capable of specialized translation. Plant cytosolic ribosomes have a half-life of 3−4 days [27], and thus building specialized ribosomes from scratch would consume much resources and time, especially considering that protein biosynthesis, including ribosome biogenesis, is the largest energy expenditure in the cell [28–30]. Consequently, *de novo* ribosome biogenesis alone may fail to satisfy the demand for cold-acclimated ribosomal complexes. Instead, we expect a mechanism that may rely on both, ribosome biogenesis and *in situ* ribosome remodelling to reshape the proteome and enhance cold acclimation.

Understanding how the plant cellular proteome is reshaped by specific translational dynamics is a demanding task. The cellular proteome is highly dynamic, and transition between different proteome states is a constant feature in the plant life cycle [31]. On top of these changes, pooling plant tissues that contain different cell-types adds a layer of complexity because cell-types feature different proteome states. For example, plant roots contain cells at different developmental and ontological stages that coexist along longitudinal and radial root axes [32]. The proteome of the root tip or root meristem is highly dynamic and differs from the longitudinal adjacent older tissue, specifically when considering protein abundance changes triggered by cold stress [14]. Root apical meristems are ‘hotspots’ of growth and require a high amount of newly synthesized ribosomes to support cell proliferation compared to the adjacent elongation and differentiation zones [33,34]. This makes root meristems an ideal system to study translational dynamics, especially those that pertain to cytosolic translation because of the relative absence of green plastids from roots. Moreover, barley roots allow sampling of a sufficiently large tip enriched for meristematic dividing cells. Sampling of the root tip and tip-adjacent root tissue instead of complete root systems avoids the pooling of highly diverse root zones and the masking of phenomena linked to rapidly proliferating cells with highly active ribosome biogenesis. Thus, barley root tips are an ideal system to understand plant translational dynamics and rapid proteome changes.

A dynamic proteome is a consequence of protein turnover, which is the balance between protein synthesis ($K_{s}$) and degradation ($K_{d}$). Translation is related to protein biosynthesis, and studying protein biosynthesis typically involves the use of stable isotope assisted mass spectrometry as a means to derive empirical measures of protein turnover [35,36]. Calculating $K_{s}$ in plants using a stable isotope pulse depends on several parameters [37,38]. The protein fraction that has taken up the externally supplied stable isotope represents the newly synthesized amount. This amount can be determined in multiple ways, for example, by isotopologue analyses of amino acids from hydrolyzed proteins [39]. Alternatively, proteomics allows for multi-paralleled isotopologue distribution analysis of peptides obtained by digestion of complex protein preparations. In both cases, the time that passes between the onset of the stable isotope pulse and sampling allows for the conversion of isotope enrichments into incorporation rates. Subsequently, several variables are required to convert isotope incorporation rates into K${,}_{s}$ rates. The first two are the total protein content and the relative growth rate (RGR), both measures of tissue growth across time. These two variables correct for differential growth and protein accumulation of analysed tissues and enable the comparison of $K_{s}$ rates between experimental systems that shift in physiology. A third set of variables that can severely bias $K_{s}$ calculations is related to the dynamics of tracer incorporation into soluble amino acid pools. Label incorporation may differ substantially between the compared plant tissues and physiological states. Label incorporation typically differs across individual proteinogenic amino acids and can change in a treatment-dependent manner [39]. Therefore, the enrichments in soluble amino acid pools must be analysed and used to predict the maximum number of potentially labelled atoms in specific peptides. These constrains are made based on peptide-specific amino acid sequences used against enrichment percentages across soluble amino acid pools. Such corrections are particularly important when the compared experimental systems are in transition between physiological states. As an exemplary case, control and cold-reared plants drastically alter total protein accumulation [14], growth dynamics [10,13,14] and pools of soluble amino acids [40,41]. Such changes, if not considered, will strongly bias observed protein $K_{s}$ rates.

In this study, we set out to uncover surmised specialized translation during cold acclimation and the mechanistic link to altered proteome dynamics in barley root proliferative tissue. We did so by tracing a ${,}^{15}$N stable isotope flux by mass spectrometry through a protein fraction enriched for large translation-related complexes. We developed a robust methodology to compare translation dynamics at non-steady-state physiological transitions in proliferating plant tissue, specifically in root preparations enriched for meristematic dividing cells. Our method corrects for differential growth and protein accumulation and our results demonstrated that these variables significantly change during cold acclimation in plant roots. Therefore, we validated the calculation of $K_{s}$ rates by using morphometric and phenotypic analyses. Importantly, we monitored label incorporation into soluble amino acid pools and demonstrated that differential amino acid accumulation is the main event of a cold-reprogrammed primary metabolome. Many of the monitored soluble proteinogenic amino acids differ significantly in ${,}^{15}$N incorporation during cold as compared to control conditions. Consequently, we corrected for the expected differential isotope tracer dilution in each specific peptide using their amino acid sequences. We adhered strictly to the physiological conditions and developmental timing relevant for germinating barley seedlings and performed our analyses in the dark prior to what would be seedling-emergence in the field and prior to the onset of photosynthetic activity. These standards enabled us to analyse physiologically meaningful cytosolic translational dynamics by calculating $K_{s}$ rates. We compared $K_{s}$ rates from plants acclimated to suboptimal temperature (4°C) with those derived from plants reared at optimal temperature (20°C) and investigated *de novo* synthesis of r-proteins in the cold-acclimated ribosome population. We found r-protein synthesis and assembly into ribosomes to be independent processes that may or may not be coordinated. In other words, cold-acclimated ribosomal complexes contained a mixture of newly synthesized and re-used r-proteins. We compared these findings to other co-purified protein complexes that are either functionally related to the protein biosynthesis machinery or have different cellular functions. Overall, our results support a model in which cold triggers alternative processing of 40S subunits through a remodelled A Small subunit (SSU) processome and triggers remodelling of 60S subunits, where specific r-proteins are preferentially synthesized and assembled. During cold, r-protein heterogeneity (substoichiometry) was concentrated at the 60S–40S interface, such that these compositional changes may be functionally correlated to the preferential synthesis and accumulation of translation initiation factors reported here. Thus, our results suggest the existence of altered translation initiation and subunit joining that may lead to selective translation. Finally, we report that cold triggered the preferential synthesis and accumulation of a sub-proteome that is functionally related to cell wall remodelling. This finding showed that the altered population of heterogeneous ribosomes produces cellular machinery needed to acclimate to cold. In summary, our work provides mechanistic hypotheses to suggest future studies towards establishing a causal link between ribosome remodelling and preferential mRNA translation during cold.

## 2. Results

### (a) The changing physiology of cold-acclimating plants leads to dynamic changes in key translational resources

Protein synthesis is central to the physiology of any organism, and the catalytic ribozymes, also known as ribosomes, synthesize all proteins throughout the phylogenetic tree of life [42], yet are functionally tuned to autocatalysis [43]. Because of this centrality, physiological changes influence and are influenced by the dynamics of ribosome biogenesis and protein synthesis. Therefore, to fully understand what types of molecular rearrangements affect protein synthesis in plants during cold, we needed to thoroughly elucidate the cold-induced shift in plant physiology and how this shift relates to key translational resources.

#### (i) Root growth dynamics

**Figure 1.**
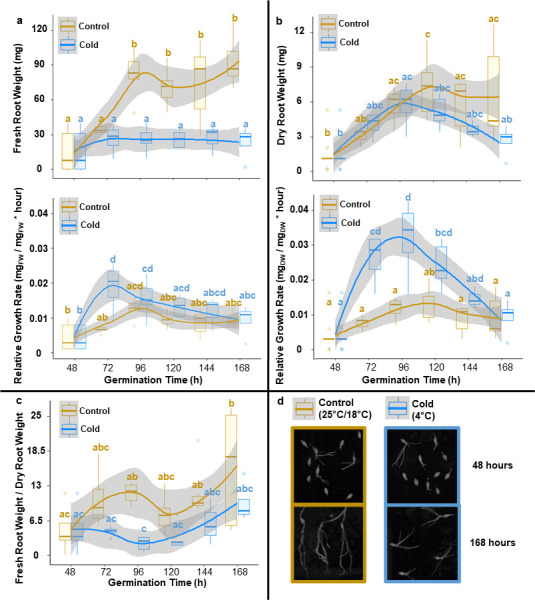
Root growth dynamics of barley seedlings reared at optimal and cold suboptimal temperatures. Related to table 1 and the electronic supplementary material, figures S1, S2 and S3. Barley seeds were imbibed and then germinated for 48 h under optimal rearing conditions. Subsequently, seedlings were transferred to different temperatures for 5 days. Panels a, b and c contain measurements of specific root growth-related variables outlined as plots wherein the means are solid-coloured lines (blue for cold and gold for control) and s.d. are shades around the mean. All of the mean values were compared by using ANOVA followed by the post hoc Tukey HSD test. The boxplots are labelled according to significant differences in the Tukey HSD test, wherein shared letters indicate a lack of significance. Starting at 48 h after imbibition, seedlings were scanned every 24 h to measure root growth with subsequent destructive harvesting to measure root FW (a— upper panel). Afterwards, root systems were dried for 70 h at 70°C and weighed again to measure root DW (b—upper panel). The recorded weights were used to assess the statistical changes in the FW-to-DW ratio during the experimental period (c). Finally, both weights were used to calculate RGRs (a and b—lower panels). DW RGR serves the purpose of normalizing protein synthesis rates to basal root growth, thus preventing biomass accumulation biases. (d) Snapshots of barley seedlings at 48 and 168 h of germination in the cold or at control temperature.

Arabidopsis rosettes delay growth for 7 days after switching to suboptimal low temperatures [10,13]. Such a growth difference can be a confounding variable when comparing the protein synthesis rates of acclimated and control plants. For example, control plants may produce more protein X (Px) than cold-reared plants; however, growth will also differ and is likely to be slower in cold-reared plants. Thus, even if cold-reared plants preferentially synthesize Px, their lack of growth would impact dry weight (DW) and protein accumulation rates, and consequently protein synthesis rates could only be correctly interpreted after normalizing to growth and biomass accumulation. To determine whether differential growth was a confounding factor in our cold acclimation experiments, we carefully phenotyped root systems of germinating barley seedlings by using non-destructive and paired destructive measurements to characterize growth across the germination period between the initial time point (*t*_0_) and the harvesting point (*t*_f_). As non-destructive measurements of growth, we used root length, diameter, volume, length × volume × 1, number of tips or branching. As destructive measurements of growth, we used fresh weight (FW) and DW (figure 1 and the electronic supplementary material, figures S1 and S2 and table S1).

FW accumulated significantly in roots from control seedlings as compared to cold-acclimated seedlings (figure 1a, upper panel). FW accumulation increased in control seedlings until around 60 h and then remained statistically constant, FW remained statistically unchanged in cold-acclimated seedlings. By contrast, DW increased in cold-acclimated and control seedlings (figure 1b, upper panel). Analysis of the FW/DW ratio (figure 1c) demonstrated a significant difference between cold-acclimating and control seedlings after 72 h and an overall trend towards a reduced ratio in the cold-acclimated seedlings. Based on these observations, we concluded that our system introduced complex dynamic changes in growth. Consequently, we determined the RGRs via FW (figure 1a, lower panel) and DW (figure 1b, lower panel) relative to root mass at *t*_f_ (see equation 8.1 in the electronic supplementary calculations).

For the RGR calculation at (*t*_0_), we set the root mass to zero. The RGR_FW_ and RGR_DW_ of the control group were constant with small, non-significant fluctuations. The RGR_FW_ and RGR_DW_ of the cold-reared seedlings significantly peaked at 60 and 72 h, respectively. We expected a decrease in RGRs owing to the transition between optimal and suboptimal temperatures. Instead, we observed a transient increase in cold-acclimated seedlings, which can be explained by the initial DW accumulation after the temperature shift and the partial compensation by fluctuating water content (figure 1). To account for the significantly different RGRs of control and cold-acclimated seedlings, we used the average RGR_DW_ over the experimental ${,}^{15}$N labelling period to normalize fractional protein synthesis rates (table 1). Because protein synthesis is one of the main contributors to DW accumulation, we chose RGR_DW_ as the most relevant correcting factor across experimental systems that differ in growth rates. DW determination is destructive and requires a significant sample mass. For these reasons, ${,}^{15}$N-label incorporation analyses cannot be directly paired and require additional replicates. Thus, we investigated the potential of non-destructive methods for RGR determination but were unable to identify a suitable replacement for RGR_DW_ (table 1).

**Table 1.** RGRs calculated from multiple root growth proxies from germinating barley seedlings.

RGR	control	cold
FW (mg mg^−1^.FW*h)	0.008	0.011
DW (mg mg^−1^.DW*h)	0.008	0.017
length per volume (cm*m${,}^{3}$/cm*m${,}^{3}$*h)	0.008	0.01
length (cm cm^−1^*h)	0.008	0.01
volume (cm cm^-3^*h)	0.008	0.008
average diameter (mm mm^−1^*h)	0.014	0.014
number of branches (#/#*h)	0.012	0.007
number of tips (#/#*h)	0.007	0.007

Averaged RGRs by root length and length × volume × 1 reflected accelerated RGR_DW_ during cold acclimation; however, none of the tested variables accurately represent the excessive transient increase in RGR_DW_ (table 1). We made a final consideration based on previous studies performed on Arabidopsis seedlings, which derived RGRs as the slope from log-linear regressions of growth over time [37]; these systems satisfied the assumption of linearity with correlation coefficients (*r*${,}^{2}$) approaching 1. For roots of germinating barley seedlings, we had to reject the linearity assumption with *r*${,}^{2}$ less than 0.5 for all of the observed variables, including root length, diameter, volume, length*volume${,}^{-1}$, number of tips or branching, as well as FW and DW (electronic supplementary material, figure S3).

#### (ii) Reprogramming of the primary metabolome

**Figure 2.**
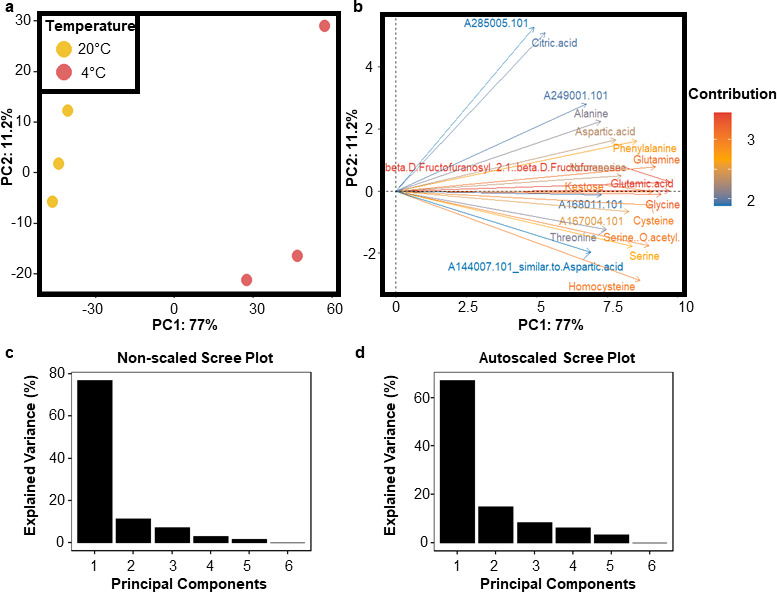
Primary metabolome dynamics in root proliferative tissue from barley seedlings reared at optimal and cold suboptimal temperatures. Related to the electronic supplementary material, table S2. The soluble primary metabolome was obtained from frozen and ground plant tissue via methanol/chloroform extraction, and the metabolite extracts were chemically derivatized by using trimethylsilyl groups to enhance volatility across the gas chromatographic column. Subsequently, the metabolome was measured in a multiplexed array by using GC–Electron impact ionization (EI)–Time-of-flight (ToF)–MS and GC–Atmospheric-pressure chemical ionization (APCI)–qToF–MS [44] in technical and biological triplicates. Metabolites were manually annotated in TagFinder, and representative tags for each metabolite were chosen. Primary metabolome data were analysed with functions of the RandoDiStats R package. (a) PCA plot, wherein metabolite features are eigenvectors determining the separation of the samples depicted as coloured dots. (b) Best 20 metabolites contributing to the separation of samples outlined in panel a by a contribution PCA plot. (c*,* d) Scree plots of cumulative variance explained in the non-scaled versus the autoscaled version of the primary metabolome PCA.

Central metabolism, which is the source of amino acid building blocks for translation, is fundamentally reprogrammed during temperature acclimation [40,41]. Therefore, before analysing the labelling dynamics of the ${,}^{15}$N isotopic flux, we decided to characterize changes in the ${,}^{14}$N non-labelled primary metabolome. To achieve an unbiased and unsupervised characterization of the primary metabolome, we used a principal component analysis (PCA) to reduce the multi-dimensionality of the entire metabolite dataset into the main orthogonal variance components (figure 2). This allowed us to identify whether the cold shift caused differential accumulation of soluble amino acids and thereby changes in the availability of essential translational resources.

By doing a targeted search for amino acids in our dataset, we found that significant changes in amino acid soluble pools were caused by cold acclimation (electronic supplementary material, table S2), and by using our untargeted PCA approach, we demonstrated that more than 70% of the variance in metabolite soluble pools is explained by cold acclimation, which is evidenced by principal component one (PC1) in figure 2a. The variance contribution of PC1 did not change with scaling (figure 2c–d), and the variance between replicates (separated in PC2) was minimal (figure 2a), which implies that there was no factor other than cold contributing to the observed change in the pools of soluble metabolites. Subsequently, we calculated the importance of individual metabolites for discriminating between cold-acclimated and control plants via a contribution plot and found that in addition to fructans and organic acids, amino acids were the main contributors to the metabolic changes caused by cold acclimation (figure 2b). The amino acids that primarily contributed to discriminating the metabolome of cold-acclimated and control plants were pyroglutamic acid (derived from glutamine converted via our extraction/derivatization procedures), cysteine, serine, homocysteine, glutamine, glycine and glutamic acid, all of which were among the top 20 log_2_-fold changed metabolites between conditions (electronic supplementary material, table S2). More generally, proteinogenic amino acids accumulated in the cold with log_2_-fold changes of 2−50 (electronic supplementary material, table S2), which suggested that the ${,}^{15}$N tracer was also likely to differentially dilute across amino acid soluble pools in cold versus control conditions. These observations highlight the need to monitor ${,}^{15}$N enrichment across soluble amino acid pools, ideally in split and paired samples with those obtained for protein or peptide ${,}^{15}$N enrichment assays.

#### (iii) Tracer incorporation dynamics into soluble amino acid pools

**Figure 3.**
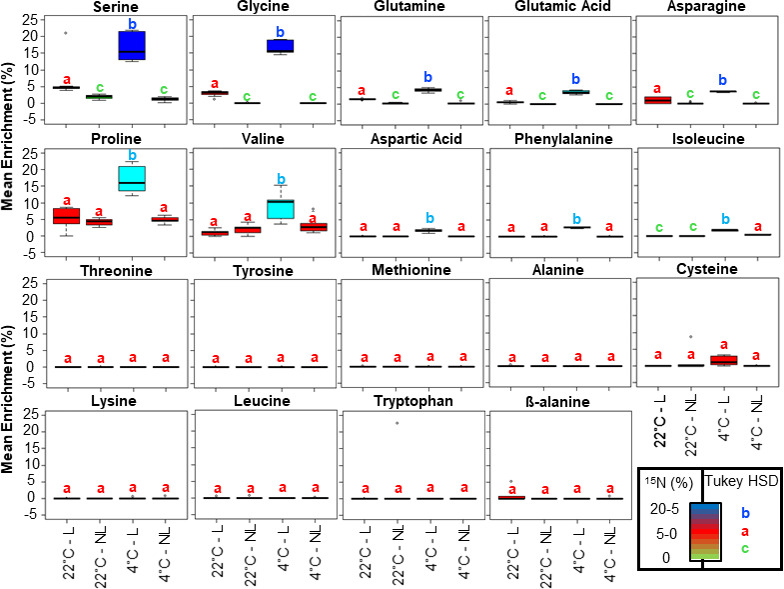
Mean isotopic enrichment of amino acid soluble pools in barley root tips from seedlings germinated at suboptimal (4°C) and optimal (20°C) temperatures. Related to the electronic supplementary material, table S3 and file S1. The soluble primary metabolome was obtained exactly as described in figure 2. Subsequently, at least three independent mass fragments per amino acid analyte, along with their isotopologue peak intensities, were extracted from total ion chromatograms. The selected fragments for ^15^N enrichment percentage calculations were those that appeared in spectra containing fewer co-eluting ions, as well as those with evidently increased mass accuracy (e.g. from the APCI platform), summing up to less noisy spectra. The fragments were corrected for natural isotopic abundance, thus enabling the calculation of enrichment percentages by using the R package IsoCorrectoR [47]. Finally, mean enrichments were statistically compared across treatments by using ANOVA, followed by a post hoc Tukey HSD test. Boxplots are coloured according to mean significant differences, where shared letters above each box indicate a lack of significant differences. NL; non-labelled and L, is labelled.

Transfer RNAs (tRNAs) get amino-acylated using amino acids present in soluble pools. After amino-acylation, tRNAs carry amino acids into a round of translation elongation by matching their anti-codon to the corresponding codon. Considering that tRNAs are extremely labile [45] and tRNA amino-acylation is difficult to measure by current methodologies [46], we used free proteinogenic amino acids as a proxy for estimating the labelling in amino acids being carried by amino-acylated tRNAs. We achieved this by monitoring the ${,}^{15}$N-tracer dynamics across soluble amino acid pools in root tips at *t*_f_ (figure 3). We supplied a mixture of equally 99% ${,}^{15}$N-labelled glycine and serine to obtain a rectangular and rapid stable isotope pulse. We chose not to use inorganic ${,}^{15}$N tracers, because these can slowly label soluble amino acid pools [35] and thereby may lead to low ${,}^{15}$N incorporation, especially under reserve mobilization conditions of a germinating seedling (cf. §3).

Consistent with our labelling strategy, soluble serine and glycine retained most of the ${,}^{15}$N tracer within root tips. Enrichments in these two targets reached 15% ${,}^{15}$N in the cold-shifted group and 2.5–5.0% in the control group (figure 3; electronic supplementary material, table S3 and file S1). Our ${,}^{15}$N enrichment values represent the experimental ${,}^{15}$N incorporation after correction for the natural isotopic abundances of the elements. The considerable differences in ${,}^{15}$N enrichment across serine and glycine pools clearly indicate that additional correction between experimental conditions is required to enhance comparisons of protein synthesis rates. We were also able to monitor 16 additional proteinogenic amino acids (figure 3), with only histidine and arginine being below the detection limit in our study. Under control conditions, glutamine (as estimated via the gas chromatography–mass spectrometry (GC–MS) profiling proxy pyroglutamic acid), glutamate and asparagine were significantly labelled at 2.5–5.0% ${,}^{15}$N enrichment, while the cold-shifted seedlings picked up 2.5–15.0% ${,}^{15}$N tracer in their roots across glutamine, glutamate, asparagine, proline, valine, aspartate, phenylalanine and isoleucine (figure 3; electronic supplementary material, table S3). The remaining monitored proteinogenic amino acids plus beta-alanine (which is a non-proteinogenic amino acid control) did not absorb ${,}^{15}$N through the re-metabolization from labelled serine and glycine. Thus, owing to the fact that even minor contributions from multiply-labelled amino acids may add up to substantial ${,}^{15}$N incorporation into peptides, the failure to account for these contributions may introduce bias into estimated protein synthesis rates. These results confirm the need to study the dynamics of the internalized tracer and to use these dynamics to correct protein synthesis rates.

### (b) Protein synthesis during transition from a physiological steady state

**Figure 4.**
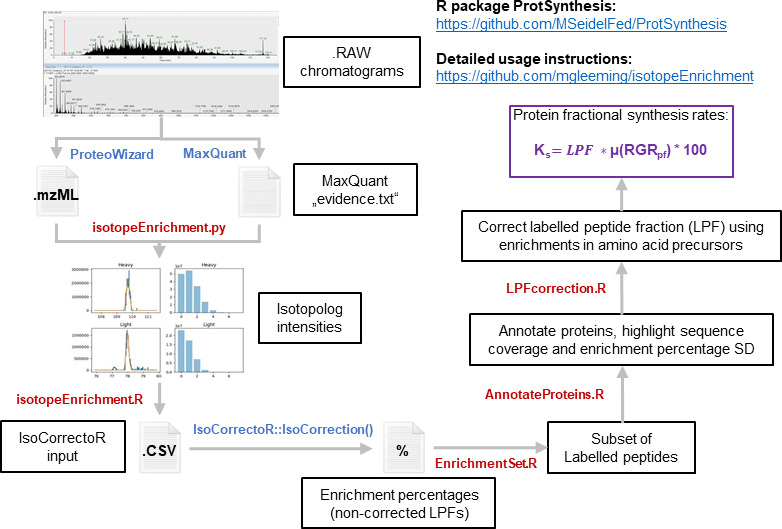
Bioinformatics workflow (python and R) enabling the calculation of fractional protein synthesis rates normalized to organismal physiology. Related to the electronic supplementary material, figures S4 and S6 and table S4. The bioinformatics pipeline is publicly available through two GitHub repositories, https://github.com/mgleeming/isotopeEnrichment, which contains detailed usage instructions for python and R functions, and the ProtSynthesis R package, which can be installed into any R environment via devtools. All of the self-written steps are outlined in red font, whereas existing algorithms that are external dependencies of the workflow are depicted in blue font. Our pipeline uses MaxQuant to locate peptides and subsequently trace back their position and recover full isotopologue intensities from .mzML files (‘isotopeEnrichment.py’ function). Subsequently, the number of isotopologue peaks is optimized for individual peptides based on the molecular formula and the enrichment percentages in soluble amino acids (‘isotopeEnrichment.R’ function). The outputs are written into files that are required by the IsoCorrectoR R package, which is used to correct for natural isotopic abundances and calculate enrichment percentages of individual peptides labelled-peptide fraction (LPF). Subsequently, statistical filters are used to identify and annotate significantly labelled peptides, as well as to derive relevant statistics that detail the quality of the protein hit by using (‘EnrichmentSet.R’ and ‘AnnotateProteins.R’ functions). Finally, LPFs are corrected using the enrichment in soluble amino acid pools (‘LPFcorrection.R’ function); with these corrected values, fractional protein synthesis rates are calculated by multiplying them by the RGR and multiplying the outcome times 100 to convert it into fractional protein synthesis rates.

In this study, we infer protein synthesis dynamics by following a stable isotope flux across soluble amino acid pools into polypeptides. The empirical calculations of isotope and metabolic fluxes are based on the assumption of a metabolic steady-state, since in such a state the metabolic targets reach a plateau of tracer incorporation, from which the differential incorporation and/or dilution of the tracer can be measured after any intervention on the system. The identification of such steady metabolic and physiological conditions for the assessment of protein turnover is no simple task. Throughout their lifespan, plants constantly transition through physiological and proteomic states that adapt to developmental and environmental factors [31]. Like many other plant systems that respond to stressors, the roots of acclimated barley seedlings did not reach an equilibrium state within our observation period. In other words, acclimation significantly altered metabolic and growth dynamics in our system in a manner that seemed to continuously change according to the measured variables (i.e. root growth and metabolite pools). Since our experimental system did not achieve a physiological steady state that would ensure that the dilution of the tracer over time from this point onwards would reflect protein degradation, we opted to determine protein synthesis rates (*K*${,}_{s}$) rather than degradation and turnover following the tracer incorporation dynamics. Our calculations consider isotopic enrichments in soluble amino acid pools, total protein contents and RGRs, to normalize individual protein synthesis rates. The details of our workflow are presented in figure 4, and all of our considerations can be found in the electronic supplementary material calculations coupled to a detailed methodological description presented elsewhere [48].

The complete workflow starting at raw data pre-processing and including the computation of all our results is provided by the ProtSynthesis R package, which contains detailed annotations and descriptions of the procedures that were used in this study [48]. This R package was implemented based on considerations made on previous plant protein turnover studies (see the electronic supplementary material calculations) [37,38].

#### (i) Synthesis and accumulation of macromolecular complexes during cold

To validate our method, we used additional and split samples to those used to analyse growth and metabolic dynamics. We obtained a ribosome-enriched complex-ome fraction from barley root tips by filtering cell lysates through a 60% sucrose cushion (SC). This ensured that only assembled macromolecular complexes were recovered from the pelleted fraction. Complex-ome total protein content was significantly higher in roots from cold-treated seedlings as compared to control seedlings (electronic supplementary material, figures S4 and table S5). We analysed label-free quantitative (LFQ) protein abundances [49,50] to determine what fraction of the complex-ome accumulated during cold (table 2). LFQ abundances were estimated with MaxQuant using the publicly available software [50]. Additionally, we monitored the incorporation of ${,}^{15}$N into individual protein components of these complexes (e.g. electronic supplementary material, figure S5) and observed 1379 good-quality peptides after applying our computational method (electronic supplementary material, figure S6 and table S4G), from which we can confidently report fractional synthesis rates. From this information, we estimated what part of the complex-ome accumulation was owing to protein synthesis and incorporated ${,}^{15}$N, or what part was owing to the lack of protein degradation and did not incorporate ${,}^{15}$N (table 2). According to the dynamics of protein accumulation and ${,}^{15}$N incorporation, we report here four types of responses for protein components of the monitored cellular complexes in table 2 (i.e. groups 1–4). Group one contains proteins that significantly accumulate owing to synthesis (incorporate ${,}^{15}$N). Group two contains proteins that significantly accumulate due to a lack of degradation (did not incorporate ${,}^{15}$N). Group three contains proteins that do not accumulate but incorporate ${,}^{15}$N (likely probably owing to high turnover). Group four contains proteins that are detected but do not accumulate nor incorporate ${,}^{15}$N. The three groups featuring significant responses, with proteins that either significantly accumulate or incorporate ${,}^{15}$N, are groups 1−3. The cold-responsive complex-ome is divided among these three groups, whereas only groups one and two are observed in the complex-ome of plant roots reared at optimal temperatures.

**Table 2.** Accumulation and origin of protein components from detected multi-protein complexes in barley root tips classified in four groups of responses during the experimental period: (group 1) accumulated and newly synthesized, (group 2) accumulated and not degraded, (group 3) not accumulated but newly synthesized, (group 4) not accumulated and not synthesized. (The table integrates the information from the A1 and G1 tabs in the electronic supplementary material, table S4 (both tabscontain their own legends to clarify the information being presented within). Gene Ontology (GO) terms have been defined in parentheses at first appearance. When cellular complexsubsets instead of the entire complex belong to a response group, the respective GO terms are indicated in parentheses).

parent categories	detected complexes	cold (4°C)	control (22°C)
cytosolic ribosome	cytosolic large ribosomal subunit (GO:0022625)	group 3	group 1
	cytosolic small ribosomal subunit (GO:0022627)	group 4	group 1
mitochondrial ribosome	mitochondrial large ribosomal subunit (GO:0005762)	group 4	group 2
	mitochondrial small ribosomal subunit (GO:0005763)	group 4	group 2
ribosome biogenesis complex	pre-ribosome, small subunit precursor (GO:0030688)	group 2 (GO:0030692)	group 4
	pre-ribosome, large subunit precursor (GO:0030687)	group 2	group 4
	small-subunit processome (GO:0032040)	group 1	group 4
	Pwp2p-containing subcomplex of 90S pre-ribosome (GO:0034388)	group 2	group 4
translation initiation complex	eukaryotic 48S preinitiation complex (GO:0033290)	group 1	group 4
	eukaryotic 43S preinitiation complex (GO:0016282)	group 1	group 4
	eukaryotic translation initiation factor 3 complex (GO:0005852)	group 1	group 4
	eukaryotic translation initiation factor 2B complex (GO:0005851)	group 3	group 4
	eukaryotic translation initiation factor 2 complex (GO:0005850)	group 3	group 4
protein folding	chaperonin-containing T-complex (GO:0005832)	group 1	group 4
ER–Golgi complex	clathrin adaptor complex (GO:0030131)	group 3	group 4
	clathrin vesicle coat (GO:0030125)	group 3	group 4
	EMC complex (GO:0072546)	group 1 (GO:0005789)	group 4
	endoplasmic reticulum exit site (GO:0070971)	group 1	group 4
	COPII vesicle coat (GO:0030127)	group 2	group 4
	trans-Golgi network (GO:0005802)	group 3	group 4
	Golgi membrane (GO:0000139)	group 1	group 4
	Golgi transport complex (GO:0017119)	group 3	group 4
	COPI vesicle coat (GO:0030126)	group 3	group 4
	exocyst (GO:0000145)	group 4	group 4
	endoplasmic reticulum-Golgi intermediate compartment (GO:0005793)	group 2 (GO:0098791)	group 4
stress-related complexes	cytoplasmic stress granule (GO:0010494)	group 4	group 4
	P-body (GO:0000932)	group 4	group 4
proteasome	proteasome core complex, alpha-subunit complex (GO:0019773)	group 3	group 4
	proteasome regulatory particle, lid subcomplex (GO:0008541)	group 4	group 4
	proteasome regulatory particle, base subcomplex (GO:0008540)	group 3	group 4
oxidative complex	peroxisome (GO:0005777)	group 4	group 4
protein degradation complex	COP9 signalosome (GO:0008180)	group 4	group 4
transcriptional regulation complex	THO complex part of transcription export complex (GO:0000445)	group 2 (GO:0000347)	group 4
	catalytic step 2 spliceosome (GO:0071013)	group 2 (GO:0097525)	group 4
nuclear complex	DNA topoisomerase type II (double strand cut, ATP-hydrolysing) complex (GO:0009330)	group 2 (GO:0030870)	group 4
	perinuclear region of cytoplasm (GO:0048471)	group 1	group 4
	nuclear periphery (GO:0034399)	group 2 (GO:0070603)	group 4
	nuclear pore (GO:0005643)	group 2 (GO:0031080)	group 4
cell cycle-related complexes	condensin complex (GO:0000796)	group 1	group 4
	MCM complex (GO:0042555)	group 2	group 4
	alpha DNA polymerase:primase complex (GO:0005658)	group 2	group 4
	DNA replication factor C complex (GO:0005663)	group 2	group 4
cell wall and membrane complex	oligosaccharyltransferase complex (GO:0008250)	group 4	group 4
	endosome membrane (GO:0010008)	group 3	group 4
	cellulose synthase complex (GO:0010330)	group 4	group 4
	endocytic vesicle (GO:0030139)	group 4	group 4
	cell wall (GO:0005618)	group 4	group 4
	plasma membrane protein complex (GO:0098797)	group 4	group 4
vacuolar complex	plant-type vacuole (GO:0000325)	group 4	group 4
	vacuolar proton-transporting V-type ATPase complex (GO:0016471)	group 3	group 4
	proton-transporting V-type ATPase, V1 domain (GO:0033180)	group 3	group 4
metabolon	oxoglutarate dehydrogenase complex (GO:0045252)	group 3	group 2 (GO:0045254)
motor-related complex	myosin complex (GO:0016459)	group 2	group 4
transmembrane complex	transmembrane transporter complex (GO:1902495)	group 4	group 4
cytoskeleton complex	microtubules (GO:0005874)	group 1	group 4
mitochondrial complex	mitochondrial outer membrane (GO:0005741)	group 1	group 4
	mitochondrial inner membrane (GO:0005743)	group 4	group 4
chloroplast complex	chloroplast membrane (GO:0031969)	group 4	group 4

The table integrates the information from the A1 and G1 tabs in electronic supplementary material, table S4 (both tabs contain their own legends to clarify the information being presented within). GO terms have been defined in parentheses at first appearance. When cellular complex subsets instead of the entire complex belong to a response group, the respective GO terms are indicated in parentheses.

#### (ii) Ribozyme-mediated proteome remodelling

Proteins from groups one and three in the cold-responsive complex-ome are the group of proteins that are preferentially translated by ribosomes during cold and thus the set of proteins that hold the potential to be the targets of specialized translation events during cold. In the following paragraphs, the results from table 2 are written in detail as well as their relationship to cold-acclimation and translational control.

The ontology term of cytosolic translation in table 2 includes ribosome biogenesis and contains protein components that belong to the three significant groups of responses (1–3). Ribosome biogenesis produces mature and translationally competent ribosomes. Therefore, tracer incorporation dynamics into these complexes can provide insights into the origin of assembled ribosomes during cold acclimation. For example, the 90S pre-ribosome in the nucleolus leads to pre-60S and pre-40S complexes. Following their maturation, pre-40S complexes are shaped by the small-subunit processome. Protein components from these four biogenesis complexes significantly accumulated at suboptimal low temperatures. Interestingly, the small-subunit processome belongs to group one during cold, i.e. it accumulated owing to *de novo* synthesis of its protein components; whereas the 90S pre-ribosome, pre-60S and pre-40S complexes belong to group two and thus accumulate owing to a lack of degradation during cold. This implies that the only potentially remodelled complex from the biogenesis subset is the small-subunit processome.

After monitoring ribosome biogenesis, we investigated the synthesis and accumulation dynamics of r-proteins assembled into ribosomes. Structural protein components of cytosolic and mitochondrial ribosomes significantly accumulate in seedlings reared at optimal temperature. Cytosolic r-proteins from both subunits belong to group one in seedlings reared at control temperature and thus significantly accumulate owing to protein synthesis. Mitochondrial r-proteins from both subunits belong to group two in seedlings reared at control temperature and thus significantly accumulate owing to a lack of degradation. By contrast, the accumulation dynamics of assembled ribosomes were altered in seedlings that shifted to cold. 60S subunit r-proteins belong to group three in seedlings shifted to cold and thus are preferentially synthesized but do not accumulate, indicating potential remodelling of 60S subunits during cold. On the other hand, the cytosolic 40S and both mitochondrial subunits do not accumulate nor newly synthesize their r-proteins during cold. Lack of observation of Chloroplastic ribosomes agrees with expectations of our plant system, because the germinating barley seedlings are non-photosynthetic and root systems should be depleted from green plastids.

After confirming that during cold cytosolic ribosomes are potentially remodelled by a newly synthesized SSU-processome and by the altered synthesis of 60S r-proteins, we monitored initiation complexes to explore the possibility of altered translation initiation being a driver of preferential translation during cold. Remarkably, translation initiation complexes preferentially accumulated only in seedlings reared at cold temperature, and all of them belonged to group one, wherein their proteins accumulated owing to *de novo* synthesis, indicating that the plant is committed to producing new translation initiation complexes, and these in turn have the potential to be remodelled during cold acclimation because some of their constituent proteins, but not all, are preferentially synthesized.

Beyond translation, we monitored protein complexes that were preferentially synthesized and accumulated in cold-reared seedlings (group one). This fraction contains the complex-ome proteome synthesized by potentially specialized ribosomes tailored for cold acclimation. We reasoned that if ribosomes preferentially translated a subset of transcripts due to their acquired structural features, we would expect to confirm that the ${,}^{15}$N-labelled proteome in cold-reared seedlings must be instrumental for successful cold acclimation. Two major ontological groups stand out as being newly synthesized and accumulating in cold-reared seedlings. In brief, cellular machinery to cope with protein misfolding and aggregation, namely chaperonin-containing TCP-1 (CCT complex) protein components and heat shock proteins, as well as cellular complexes that mediate the remodelling of cell walls and cellular membranes. Both types of complexes align well with the cellular physiology of cold acclimation, and thus these results prompted us to conduct in-depth analyses of the extent of triggered ribosome heterogeneity and its potential structural link to the observed proteome shift that occurs owing to protein synthesis by ribosomes.

### (c) Recycled and newly synthesized r-proteins associate with ribosomes during cold acclimation

The existence of a sub-proteome specifically synthesized by ribosomes in the cold prompted us to thoroughly investigate ribosomal protein heterogeneity, both in terms of r-protein stoichiometry and r-protein synthesis. As we demonstrated before, the spatial distribution of r-protein heterogeneity can be correlated with specialized translational functions [12,51]. Therefore, the different layers of r-protein heterogeneity afforded by our data are a robust reflection of potential ribosome structural adaptation during cold acclimation in plants.

**Figure 5.**
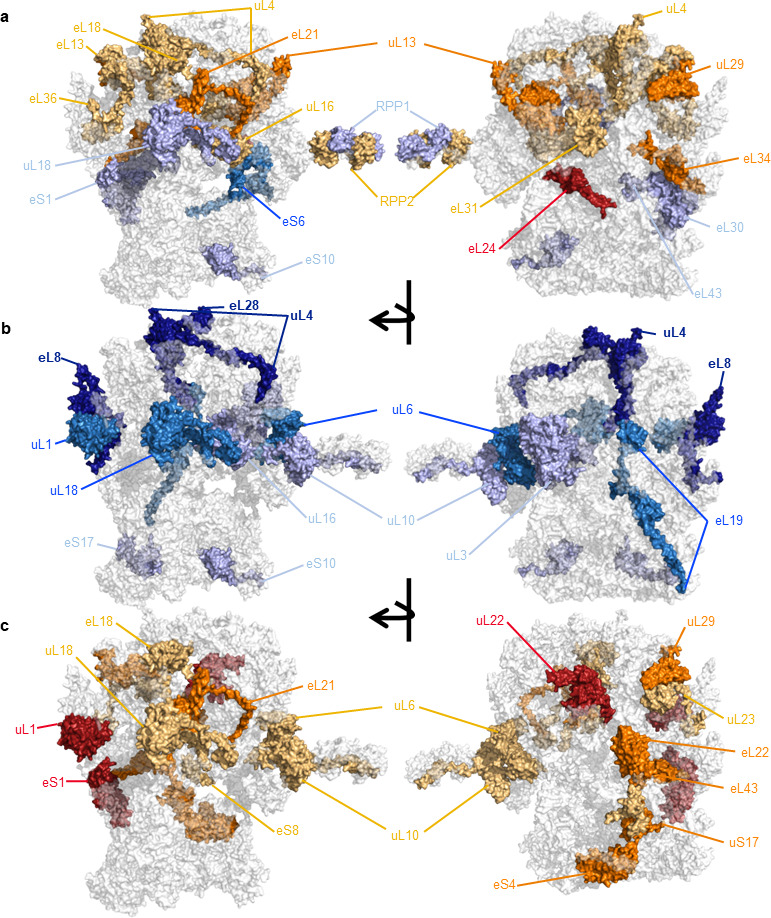
Characterization of heterogeneous barley ribosomes, their ribosomal protein (r-protein) composition, fractional synthesis rates and induced substoichiometry during canonical germination at optimal temperatures or after a shift to cold suboptimal temperatures. Related to electronic supplementary material, figures S5 and S7 and table S4. Barley ribosomes from root ${,}^{15}N$-labelled proliferative tissue were purified and used to profile the r-proteome. Abundances and isotopologue envelopes were recovered from the mass spectrometry data and used to calculate the average ribosome relative stoichiometry of r-proteins and their fractional synthesis rates. (a) r-protein substoichiometry; in light-orange, orange and red are r-proteins significantly accumulated at optimal temperatures with *p*_adj_ values *<0.1, **<0.05 and ***<0.01, respectively. In light-blue and blue are r-proteins significantly accumulated during cold in the ribosomal population with *p*_adj_ values *<0.1 and **<0.05, respectively. (b) Preferential synthesis of assembled r-proteins during cold acclimation. In light-blue, blue and dark-blue are r-proteins preferentially synthesized and assembled during cold in the ribosomal population with *p*_adj_ values *<0.1, **<0.05 and ***<0.01, respectively. (c) Preferential synthesis of assembled r-proteins under controlled temperature. In light-orange, orange and red are r-proteins significantly accumulated at optimal temperatures with *p*_adj_ values *<0.1, **<0.05 and ***<0.01, respectively. Substoichiometry and preferential synthesis refer to peptides with significant changes in peptide abundances or their fractional synthesis rates, respectively.

To characterize cold-induced r-protein heterogeneity and to attempt to decipher its origin and potential for altered functionality, we adjusted protease digestion to the requirements of a highly basic ribosomal proteome ([48]; figure 3) and profiled ribosome-enriched barley root tip extracts. To validate that our method enabled the recovery of native ribosomes, we subjected *Escherichia coli* 70S ribosomes to the same purification method and assessed the completeness of the recovered ribo-proteome. We observed 21/21 30S SSU r-proteins and 33/33 50S large subunit (LSU) r-proteins. These measurements demonstrated reproducible abundances in triplicate measurements [48]; figure 4). We then calculated r-protein abundances, relative stoichiometry and fractional synthesis rates for assembled r-proteins from barley root tip extracts at the physiological transition from optimal to suboptimal low temperatures (figure 5; electronic supplementary material, table S4H).

We report LFQ intensities for 17 r-protein families from the small 40S subunit (SSU) and 38 r-protein families from the 60S LSU, all bound to ribosomal complexes (electronic supplementary material, table S4C). These are regarded as 55 high-confidence r-protein family annotations, defined as having at least one paralogue gene with multiple unique peptides. High-confidence annotations included a total of 95 paralogue genes identified. From the identified paralogues, we observed that the sum of SSU r-protein abundances correlated linearly by *r*${,}^{2}$ = 0.98 with the sum of LSU r-protein abundances across temperatures, maintaining a constant ratio of 3x LSU relative to 1x SSU (electronic supplementary material, figures S7 and table S4C). This observation implies that the number of assembled LSU and SSU subunits is co-regulated, especially considering that there were more LSU and SSU r-proteins (assembled subunits) in seedlings reared at control conditions, and yet the ratios were maintained.

Previously, we reported that r-protein abundances, on average (i.e. considering both assembled and non-assembled r-proteins), increased in barley root tips of germinating seedlings subjected to cold [14]. Here, we report that cold-reared seedlings featured fewer assembled ribosomes as judged by the summed abundances of 40S and 60S r-proteins. Importantly, in table 2, we report that during cold there is significant accumulation of ribosome biogenesis and translation initiation complexes. Thus, taken together, our previous and current data imply that upon shifting seedlings to cold, r-proteins accumulate significantly above the level of control, but their localization is probably constrained to the yet non-assembled fraction of r-proteins or to small immature ribosomal complexes that have not entered the translational pool yet.

#### (i) Ribosomal protein substoichiometry

Control seedlings contained more assembled 40S and 60S subunits in their root tips. Consequently, to avoid biases when calculating r-protein substoichiometries, we used the sum of 40S proteins to normalize individual r-protein abundances of the SSU and the sum of 60S proteins to normalize individual r-protein abundances of the LSU. In this way, we corrected for the relative number of individual subunits across samples, as we have done previously [13]. After normalization we analysed the low-temperature induced r-protein substoichiometry (figure 5a).

We used carefully aligned HORVU sequences to determine the closest identity of HORVU r-protein paralogues with respect to Arabidopsis (see §4g(ii) for details on the alignment and the electronic supplementary material, table S4H for detailed information on paralogue identity, substoichiometry and synthesis rates), each HORVU code represents a single r-protein paralogue match and is also used as an identifier of its respective r-protein family when appropriate. The population of 40S subunits was enriched in four r-protein paralogues during cold, which included eS10, eS1 and eS6. Thus, the population of 40S subunits in barley root tips is not canonically complete; these paralogues are absent compared with the cold population. Likewise, the population of 60S subunits contains paralogues that are both relatively depleted or accumulated during cold. Some r-protein families are depleted during cold: P2, uL16, eL13, eL18, eL31, eL36, uL4, eL34, uL29, eL21, uL13 and eL24. Some r-protein families accumulate during cold: P1/P2/P3, eL30, eL43 and uL18.

#### (ii) Altered ribosomal protein synthesis and ribosome remodelling

We next examined r-protein synthesis rates, inferred from peptide isotopic envelopes (electronic supplementary material, figure S5), to understand which r-proteins were newly synthesized (figure 5b,c). In general, the cold-induced changes in r-protein synthesis did not coincide with substoichiometry, implying independence between r-protein synthesis and ribosome assembly or remodelling. In terms of preferential synthesis of r-proteins at optimal rearing temperatures, 40S subunits were enriched in four newly synthesized r-protein paralogues (figure 5c), which included eS8, uS17, eS4 and eS1. Similarly, 40S subunits at cold-rearing temperatures were assembled or remodelled using two newly synthesized r-protein paralogues, which included eS10 and eS17.

In a similar manner, 60S subunits were assembled or remodelled using several newly synthesised r-protein paralogues that were significantly synthesized either during optimal rearing temperatures or during cold. Some paralogues were preferentially synthesized and assembled at optimal rearing temperatures: uL10, eL18, uL23, uL18, eL6, eL21, eL22, uL29, eL43, uL22 and uL1. Some paralogues were preferentially synthesized and assembled during cold: uL16, uL3, eL19, uL1, uL18 eL6, eL28, uL4 and eL8.

#### (iii) Ribosomal protein synthesis and accumulation dynamics in assembled ribosomes

Based on our results, five types of paralogue-specific or generalized r-protein family associated phenomena and synthesis dynamics can be deduced:

- **paralogue switches:**
  — based on r-protein synthesis, uL1 paralogues were switched during cold and control-rearing temperatures;
  — based on r-protein substoichiometry, P1/P2/P3 paralogues were switched during cold and control-rearing temperatures;
- **families with paralogues sharing ribosome-bound accumulation or synthesis dynamics:**
  — based on r-protein synthesis uL18 and uL1 r-protein families shared cold-specific dynamics among paralogues;
  — based on r-protein substoichiometry, eL24 and uL13 r-protein families shared control-temperature-specific dynamics among paralogues, while eS1 r-protein family shared cold-specific dynamics among paralogues;
- **paralogue splice variants (peptides from the same protein with different synthesis dynamics under cold and control conditions):**
  — uL10 featured one unique peptide from exon 5 that was preferentially synthesized during cold-rearing temperatures and one from exon 4 during control-rearing temperatures;
- **specific paralogues that share ribosome-bound accumulation and synthesis dynamics:**
  — eS10, eL18, eL21, uL29, uL18;
- **specific paralogues with inverse ribosome-bound accumulation and synthesis dynamics:**
  — accumulated during control-rearing temperatures in ribosomes but preferentially synthesized during cold: uL16, uL4;
  — accumulated during cold-rearing temperatures in ribosomes but preferentially synthesized in the controls: eS1, eL43.

## 3. Discussion

### (a) Morphological phenotype of barley roots during low-temperature germination

Plants arrest growth during the first week of cold acclimation [10,13,14]. Arabidopsis roots reduce mitotic division (but not cell elongation) at 4°C, which reduces meristem size [52]. Cold-acclimated barley roots reduce protein content [14]. In our hands, root length and volume increased in controls probably owing to water accumulation (electronic supplementary material, figure S2), as shown by differences in FW (figure 1a). Conversely, root DW did not differ between cold and control, and thus the fresh to DW ratio significantly changed. Therefore, we conclude that global analyses of protein turnover need to account for this difference.

### (b) Metabolic phenotype of cold-acclimating barley roots and links to translational responses

After 2 days of cold (4°C) in the dark, glucose increases in barley, whereas proline, sucrose and total lipids decrease [53], which coincides with the peak of DW accumulation that we report. Transcriptional studies on the third day of cold predict the accumulation of sugars and polyols such as maltose, glucose, trehalose and galactinol [54]. We showed that glucose and sucrose accumulated on the fifth day of cold. Probably owing to differences of non-photosynthetic root and photosynthetic leaf tissue, maltose did not accumulate, and galactinol and trehalose were not detectable. Amino acid biosynthesis was not predicted to be transcriptionally upregulated [54], and yet we observed that 25 out of the 29 detected amino acids, including the osmoprotectant proline, accumulated during cold. Amino acid pools increase in germinating seedlings owing only strategy to monitor isotopic to mobilized nitrogen resources from seed storage proteins in alignment with their non-photosynthetic, heterotrophic physiology. Nonetheless, we demonstrated that some amino acids incorporate ${,}^{15}$N from externally fed ${,}^{15}$N-serine and ${,}^{15}$N-glycine. Thus, amino acids can also be newly synthesized and complement mobilized resources. Control mechanisms involving amino acid mobilization, transport to and uptake, *de novo* synthesis and consumption may be controlled by translational activity during cold since there are no transcriptional predictions. The role of soluble sugars, here glucose and sucrose, and amino acids, specifically proline, during cold is thought to be that of osmoprotectants, i.e. compounds that stabilize proteins and membranes and thereby contribute to cold and freezing tolerance [55]. The role of accumulated metabolites may also be linked to generating feedback for the global regulation of translation and growth [56].

### (c) Amino acid metabolism and ${,}^{15}$N isotopic flux

Nitrogen nutrition in germinating barley determines the best strategy for isotopic flux studies. Most nitrogen used by barley embryos originate from degraded storage proteins located in the endosperm [57–60]. Nitrogen is transported into the embryo and from there to proliferating root tissue. Nitrogen transport and re-assimilation are fundamental for gene expression programmes in barley caryopses [61]. Germinating barley embryos activate genes involved in the biosynthesis, metabolism and transport of amino acids at an early stage, 2–3 days after germination [62], and peptide transporters are considered to be particularly critical for normal germination [63]. At the proteome level, nitrogen mobilization systems are induced and activated [64]. Proteases, including carboxypeptidases and aminopeptidases, provide peptide- or amino acid-substrates that are released, transported and consumed during germination [62,65–67]. Similarly, just prior to radicle sprouting, proteins involved in amino acid biosynthesis and transport are upregulated, whereas those involved in amino acid catabolism are unresponsive [57], suggesting that nitrate reductase is not required for nitrogen-assimilation. After feeding ${,}^{15}$N-labelled serine and glycine, the spread of ${,}^{15}$N across proteinogenic soluble amino acid pools was limited but increased during cold. Thus, our data support the previous conclusion that amino acid degradation and re-assimilation are suppressed processes during barley germination [57]. Therefore, the use of labelled amino acids to introduce a tracer into germinating seedlings is probably the only strategy to monitor isotopic fluxes into newly synthesized protein. Germinating barley seedlings have at least four systems for amino acid uptake [68], all of which depend on protein hydrolysis products being taken up into the scutellum for utilization [69]. These paths ensure that the exogenous supply of ${,}^{15}$N-labelled amino acids is introduced into and used by the plants. Owing to the availability of endogenous amino acid resources from the seed, incorporated ${,}^{15}$N gets diluted. At low temperatures, the enzymatic activities and cellular dynamics slow down. Therefore, the mobilization of amino acids and peptides for nitrogen supply is also affected. Accordingly, cold-acclimating seedlings take up more exogenous labelled amino acids and spread them across soluble pools. A nitrogen deficiency resulting from reduced amino acid mobilization may be compensated by an increased uptake of available nutrients through amino acid and peptide transporters that are already expressed during germination. Owing to the cold-induced spread of ${,}^{15}$N from ${,}^{15}$N-serine and ${,}^{15}$N-glycine, a careful numerical correction must be made to study protein synthesis by considering ${,}^{15}$N enrichment across amino acids used against peptide compositions. In other experimental systems, for example, when studying photo-autotrophic tissue, photorespiration or the one-carbon-metabolism in plants, ${,}^{15}$N incorporation can be performed using nitrate or ammonium salts. These inorganic nitrogen sources prevent altering specific pools of soluble amino acids [70].

### (d) Ribozyme-mediated ${,}^{15}$N incorporation into protein

${,}^{15}$N-labelled amino acids conjugate with tRNAs and are transported to ribosomes, where they enter the elongation cycle and end up as a monomer within a newly synthesized polypeptide [71]. Amino acids exist as soluble pools and are loaded onto aminoacyl-tRNAs, which are present in much lower proportions. tRNAs are also labile and their turnover is extremely rapid [45], suggesting that ${,}^{15}$N isotopic enrichment in soluble amino acid pools is a valid proxy for the ${,}^{15}$N enrichment of aminoacyl-tRNA conjugates.

The main function of ribosomes is autocatalysis [43], and the cellular proteome within one degree of ribosomes comprises macromolecular complexes [42]. By purifying complex-ome, translation-related multi-protein complexes are recovered while co-purifying other relevant cellular complexes for comparative purposes. In this manner, we tested the link between surmised functional ribosome heterogeneity and altered rates of protein synthesis during cold. Altered protein synthesis can be caused indirectly, for example, via transcript recruiting mechanisms that are independent of the ribosome structure [72], or by direct translational control [73]. Direct translational control implies an altered and selective ribozyme function that shapes the proteome. In our system, translation during cold is performed by a ribosomal population that is heterogeneous and substoichiometric in its r-protein composition (figure 5). Altered r-proteome compositions confer to many metazoan ribosomes the ability to selectively recruit transcripts for translation, i.e. to specialize [17]. Compared to higher metazoans, plants have an outstandingly increased number of r-protein paralogues [74], and these gene duplications lead to novel and divergent functions [75]. Thus, it is conceivable that specialized paralogues may equip heterogeneous ribosomes with properties to perform direct translational control and/or efficiently adapt them to be functional at cold temperatures. These possibilities remain to be formally tested in the context of cold acclimation and translational efficiencies [76]. Our results represent a step forward that demonstrates which structural adaptations may confer to plant ribosomes the ability to shape the proteome by conducting specialized translation.

### (e) Translational dynamics of heterogeneous ribosomes

In Arabidopsis, cold-heterogeneous translating ribosomes exhibit r-protein substoichiometry around the polypeptide exit tunnel (PET), with many of the differential r-proteins being removed during cold [13]. Herein, we report that barley ribosomes also exhibit subtractive heterogeneity [77]. In the cold-ribosomal population, uL4 and uL29 are substoichiometric (figure 5). The protein uL4 localizes within ribosomes adjacent to uL29 and is essential for PET assembly [78–81]. Internal loops of uL4 form constriction sites for nascent polypeptides [82]. Assembly of the PET is particularly critical during cold for both yeast [83] and Arabidopsis [11], which share a homologous 60S maturation factor that when knocked out leads to cold sensitivity, namely Rei1 in yeast and REI-like 2 in Arabidopsis. Rei1 inserts its C-terminus into the PET to check the integrity of the tunnel as a quality control step before making 60S subunits translationally competent [84]. Subtractive r-protein heterogeneity near the tunnel could be indicative of ribosomal RNA (rRNA) disorder, a defective tunnel assembly and/or altered PET structures. All three effects may cause a demand for PET quality control during cold. We estimate that a typical ratio of 60S : 40S subunits in barley is 3 : 1 (electronic supplementary material, figure S7), suggesting that surplus LSUs may be disposed of without compromising the minimal translational stoichiometry of 1:1 (LSU : SSU). LSUs with a faulty PET may be an important subset of the cellular LSU population that require a checkpoint and an exclusion mechanism to efficiently select non-defective 60S subunits for the translationally active ribosome pool or that are used to translate specific transcript subsets.

Both 60S r-proteins that accumulated in assembled ribosomes during cold are located near important inter-subunit bridges, namely uL18 and eL30 [51,85]. Similarly, the population of 40S subunits accumulated eS6 and eS1, which also form inter-subunit bridges that connect 40S and 60S subunits [51,85]. Another r-protein that was more abundant in 40S subunits is eS10, which links the large uS3 hub containing the ribosomal region adjacent to the tRNA–mRNA entry sites with the uS13–uL11 inter-subunit bridge [51]. Bridges between subunits in bacteria have been shown to directly affect initiation factor-dependent translation [86]. Our observations suggest that cold-triggered r-protein heterogeneity is focussed on inter-subunit connectivity and may influence joining of 60S subunits with initiation complexes. Indeed, we report the preferential synthesis and accumulation of translation initiation complexes during cold. These findings in combination, substantiate a hypothesis of altered initiation dynamics being functionally related to r-protein heterogeneity.

### (f) Translation initiation: newly synthesized complexes

Translation initiation complexes accumulated during cold owing to synthesis of their protein components. Accumulation and *de novo* synthesis imply control over the type of complexes that participate in ribosome-transcript selection. Translation initiation is a highly conserved, sequential process [87–95], that has plant-specific adaptations [96]. The significant components of the initiation machinery during cold are given below:

- eukaryotic translation initiation factor 3 subunits A, B, C and E (eIF3A–C, E). The eIF3 complex consists of 13 subunits (A–M) and is the largest and most complex initiation factor in eukaryotes [97,98]. The complex is associated with pathological conditions in higher metazoans [99]. Subunits A and C link eIF3 to 40S subunits via the platform on the solvent side [89], while subunit E interacts with eIF1 and eIF4G [100]. Accumulated and *de novo* synthesized eIF3 subunits serve as anchors between ribosomes and mRNA recruitment factors. Subunit B is part of the eIF3b-i-g module and presumably interacts with the 40S subunit directly at the mRNA entry site by occupying the entry channel [101]. The accumulation of said subunits is associated with cancer in humans via increased [102–104] and selective [105,106] translation. We argue that eIF3 may alter transcript–ribosome association dynamics during cold in association with cold-induced r-protein heterogeneity, enabling mRNA selectivity; and
- eukaryotic translation initiation factor 2 subunit 1 (eIF2α). The factor eIF2α catalyses the first step of 40S to initiator–tRNA (Met-tRNA) association [107] and is the central element of the integrated stress response in eukaryotes [108]. It causes global decreases in protein synthesis after phosphorylation and promotes selective translation of protein products required for survival [109]. Plants may operate a conserved version of this robust stress response as part of successful acclimation to cold.

The accumulation of a protein fraction enriched in r-proteins during cold (previously reported by us [14]), and demonstrated again in this study (electronic supplementary material, figure S4), originates from ribosome biogenesis and translation initiation complexes. These observations highlight the importance of translation initiation during cold and indicate a potential functional role for substoichiometric subunits (figure 5). The accumulation of pre-ribosomes and translation initiation complexes, along with a maintained ratio between 40S and 60S subunits, suggests that the limiting step during cold acclimation is initiation. We hypothesize that the set of competent 60S subunits assembled during cold may select which transcript will be translated. This hypothesis is based on the observed excess of PICs and ICs. We do not rule out that pre-initiation complex (PIC)- and initiation complex (IC)-assembly may generate an additional selectivity layer that may not be associated with r-protein heterogeneity.

### (g) Ribosome biogenesis: assembled and remodelled ribosomes

Ribosome biogenesis is connected to major environmental and developmental responses [110]. We found that ribosome biogenesis complexes accumulated during cold either owing to slowed-down processing or owing to lack of protein degradation because these complexes did not take up the ${,}^{15}$N tracer. The only pre-ribosomal complex that accumulated newly synthesized protein components was the SSU processome; the earliest pre-40S complex identified in eukaryotes. The SSU processome uses accessory factors to initiate, process and mature 40S subunits [111]. Newly synthesized protein components may indicate alternative assembly of the SSU processome during cold, which could lead to heterogeneous 40S subunits, as those presented in our results (figure 5). Furthermore, there were fewer assembled ribosomes during cold, and yet specific 60S components were preferentially synthesized, to such an extent that the 60S GO term was enriched among the cold-synthesized proteome. Thus, taken together our data suggest that there is LSU restructuring owing to altered r-protein synthesis and incorporation into cold-acclimated 60S LSU, while the r-protein stoichiometry of 40S SSUs changes probably as a function of an adapted SSU processome. Both layers of r-protein heterogeneity are complemented by increased abundances of ribosome biogenesis complexes, which suggests that ribosome heterogeneity could arise in part from the ribosome assembly line. We cannot rule out that part of the newly synthesized r-proteins in ribosome complexes may arise from r-protein replacement or exchange mechanisms [112].

Considering the synthesis of individual r-proteins, where only a subset incorporated ${,}^{15}$N, ribosomes are assembled or remodelled by using new and previously synthesized (recycled) r-proteins. As variability in r-protein synthesis must match that of degradation, our observation confirms previous reports of plant cytosolic r-proteins exhibiting the greatest variability in degradation rates among protein components of large cellular complexes [38]. This variability makes ‘economic’ sense, as ribosome assembly and protein biosynthesis impose the greatest cellular costs [113]. Therefore, continued synthesis of every component within the translational apparatus would be detrimental, if previously synthesized r-proteins are available and re-usable. Ribosomal proteins in plants have a half-life of approximately 4 days [27], and given the slower cell dynamics at cold temperatures, a sole reliance on ribosome biogenesis to control translation would be too slow. Therefore, remodelling pre-existing ribosomes to adapt their function could be an efficient way to acclimate. In higher metazoans, ribosomes in the neuropil, which are located distant from the nucleolus, are remodelled *in situ* [114]. Thus, ribosome remodelling is able to regulate local protein synthesis depending on the subcellular location [114] and is also a means to respond quickly to stress [112,115]. Whether plants operate similar remodelling mechanisms, remains to be tested. We do not report rRNA synthesis rates, which is required to determine whether a complete biogenesis cycle generated heterogeneous ribosomes or whether on the contrary heterogeneous ribosomes resulted from remodelling.

### (h) Translational outcome of heterogeneous ribosomes: a proteome shift

Remodelled heterogeneous ribosomes directly or indirectly incorporate ${,}^{15}$N, affecting the synthesis and accumulation of protein folding machinery, complexes from the endoplasmic reticulum (ER) and Golgi, nuclear complexes, complexes related to the cell cycle and cell wall, microtubule complexes and protein complexes of the outer mitochondrial membrane. Many of these complexes are of great importance for acclimation, evidenced not least by the fact that the plant accumulates them even though resources are limited in the cold.

Six out of eight subunits from the cytosolic chaperonin T-complex-protein 1 ring complex (CCT) are preferentially synthesized during cold, leading to its accumulation. CCT promotes the folding of newly synthesized polypeptides [116,117] or their aggregation and subsequent degradation [116,118,119]. During cold, the PET is remodelled, and the signature of a defective tunnel is protein misfolding [82,120,121]. It is, therefore, conceivable that an altered tunnel leads to increased protein misfolding. Such misfolding may be counteracted by synthesizing more CCT complexes. We also found that several heat shock proteins are preferentially synthesized and accumulated during cold. The function of heat shock proteins underscores the hypothesis that protein misfolding is an urgent problem that cold-acclimating plants need to address.

Nuclear and cell cycle complexes, including condensin, were preferentially synthesized and accumulated during cold. Condensin promotes chromosome assembly during the cell cycle but also regulates gene expression [122,123]. Plants stop mitotic activity during cold [52]. Therefore, condensin is less likely to accumulate to promote cell cycle progression but may accumulate to promote transcriptional control.

All of the other preferentially synthesized and accumulated complexes can be classified into machinery that transports or targets cellular membranes and the plant cell wall. Among these, we observed ER and Golgi components, as well as cell wall and microtubule proteins. These proteins function as transporters, glycosyltransferases or are part of the cell wall structure. The integrity of cell membranes and walls is threatened during cold, as membrane fluidity decreases and dehydration increases [124]. Physical changes such as remodelling of the membrane lipidome [125] are essential to survive cold periods [126], and specific lipid species confer cold resistance to cereals [127]. Membrane remodelling relies on enzymatic activity to synthesize new lipid components [128] and vesicle transport or membrane junctions to distribute them among the cellular membrane. New protein components are also distributed and transported as vesicle cargoes to the cell periphery. The secreted proteins are synthesized at the ER and Golgi membrane compartments, where the shuttles that transport them are ready to deliver them to their site of function. We provided evidence that the transport machinery is active during cold, accumulates and is newly synthesized. This effect is part of the plant’s cold response and may attempt to mitigate the cold-induced increase of membrane rigidity and loss of membrane integrity.

Cold-heterogeneous ribosomes also synthesize components of other macromolecular complexes without accumulating them. This mode of synthesis implies the potential remodelling or replacement of proteins that continue to be present in equal amounts owing to continued turnover. Proteins that fall into this category include components of 60S ribosomal subunits, translation initiation complexes, ER–Golgi components, proteasome, vacuolar proton-transporting ATPase and the oxoglutarate metabolon.

The translation of all of these sub-proteomes indicates that cold-heterogeneous ribosomes directly or indirectly control their translational output to efficiently acclimate plants to cold. Therefore, our study offers testable hypothesis to eventually answer to what extent the remodelled ribosomal population characterized in this work represents functional ribosome heterogeneity and specialization during temperature acclimation in plants.

## 4. Material and methods

All electronic supplementary material associated with this manuscript can be accessed via Figshare https://figshare.com/articles/dataset/Supplemental_Files/25923664. 

### (a) Experimental design

**Figure 6.**
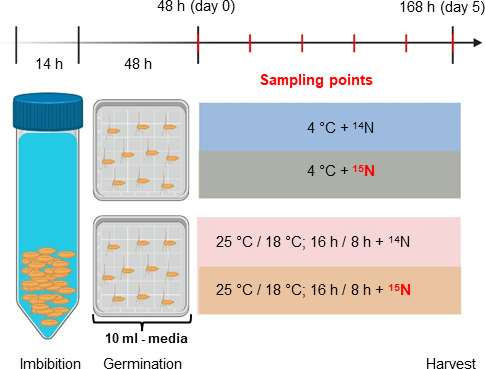
Experimental set-up. Related to the electronic supplementary material, figures S8 and S9. *Hordeum vulgare* seeds were soaked in sterile H_2_O for 14 h of imbibition. Seeds were transferred to plates for germination for 48 h. At 48 h (considered experimental time zero), treatments were applied. Half of the seedlings were treated with media supplemented with ^15^N compounds, and the other half supplemented with ^14^N compounds as a control. Half of the seeds were shifted to 4°C to induce cold acclimation. The other half remained in the control growth chamber with an optimal temperature fluctuation of 25°C for 16 h and 18°C for 8 h. Six seedlings were harvested daily per treatment after time 0 for phenotypic analyses. After harvest, each seedling was scanned for phenotyping, and roots were weighed for FW, dried for 70 h at 70°C, after which they were weighed again for DW. For primary metabolome, protein synthesis and accumulation analysis, root tips were collected in 1.5 cm segments on the fifth day of acclimation (considered the final experimental time). Created with BioRender.com

We followed previously published procedures [14] to ensure legitimate, physiologically relevant rearing conditions for barley seedlings (figure 6). We imbibed seeds for 14 h under sterile conditions and then transferred them for germination on plates with a small addition of nutritious liquid medium for 48 h. At this time, we applied different temperature regimens to mimic an agronomically relevant temperature drop after sowing barley in the field. Germination occurred in the dark for an additional 60 h, which corresponds to the time lapse it takes for a barley seedling to germinate when planted at an optimal seeding depth [129].

Control seedlings germinated at an optimal temperature of 25°C/ 18°C—day/night regimen completely covered in the dark. Barley seedlings shifted to cold temperatures and were cultured at a suboptimal temperature of 4°C. Differential growth, which is one of the necessary variables for calculating protein synthesis rates, was monitored by using high-resolution imaging of independently germinated seedlings every 24 h (electronic supplementary material, figure S8). Throughout the 5 day period, etiolation, premature greening, seed nutrient starvation and other processes resulting from prolonged darkness were monitored to ensure physiological legitimacy [129]. Seedlings at 4°C developed more slowly but showed no other macroscopic phenotypic changes associated with prolonged darkness (electronic supplementary material, figure S8). We began labelling seedlings with ${,}^{15}$N coincident with the temperature shift at 48 h after germination (*t*_0_) and throughout the temperature treatment to ensure that the dynamics of tracer incorporation reflected the physiological changes associated with cold acclimation and delayed growth. After 108 h of germination, i.e. at the last experimental time point of the labelling experiments (*t*_f_), the root tips of the seedlings were harvested to analyse the isotope incorporation and pool sizes of soluble amino acids and to measure the relative abundances and ${,}^{15}$N enrichment of individual proteins (electronic supplementary material, figure S9). Non-labelled controls were supplemented with the same compounds containing ${,}^{14}$N atoms.

### (b) Plant rearing

#### (i) Surface seed sterilization and imbibition

*Hordeum vulgare* cultivar Keel seeds were obtained from the University of Melbourne in previous studies [130]. Seeds were placed inside sterile 50 ml Falcon tubes (max 2 g per Falcon tube with approx. 40 seeds) amounting to a total of approximately 600 seeds (i.e. 15 Falcon tubes). The non-biological materials were surface sterilized with 70% ethanol and placed inside a clean bench, followed by UV sterilization. The seeds were soaked in 70% ethanol and shaken gently for 1 min, and the ethanol was then discarded. A 1% bleach solution (0.042% sodium hypochlorite) was subsequently added to the Falcon tube and gently shaken for 10 min, after which the bleach solution was discarded. The seeds were rinsed five times with sterile Milli-Q H${,}_{2}$O and gently shaken for 5 min each time to completely remove the hypochlorite, after which the water was discarded. After straining the water, the seeds were soaked in sterile Milli-Q H_2_O, and the Falcon tubes were wrapped in aluminium foil to prevent any light exposure for 14−18 h, half of this time the growth chamber was maintained at 25°C and the other half at 18°C, in order to mimic the optimal daily temperature fluctuations and thereby initiate imbibition of the seeds.

#### (ii) Seedling germination and treatment

Seeds were germinated and treated in complete perceived darkness by using a green light filter to cover the light entering the clean bench where the seeds were transferred to plates. For germination, seeds were transferred to Petri dishes that were filled with 10 ml non-labelled, non-supplemented Scheible medium [131] for 48 h; moreover, 8−16 seeds were transferred into each Petri dish. The dishes were sealed with micropore tape, wrapped in aluminium foil and placed in a phytotron growth chamber (Weiss Technik, Germany) with temperature settings of 25°C for 16 h and 18°C for 8 h until the completion of 48 h to allow for germination. When 48 h had passed, six germinated plants from a Petri dish were harvested and processed to calculate RGR at time point zero (explained below). Afterwards, working on the clean bench and under a green light filter, the medium in the dishes was disposed and exchanged for ${,}^{15}$N-labelled and ${,}^{14}$N-non-labelled supplemented media. Namely, 42 dishes were filled with 10 ml non-labelled Scheible medium supplemented with 0.5 mM ${,}^{14}$N serine and ${,}^{14}$N glycine, and 30 dishes were filled with 10 ml labelled Scheible medium supplemented with 0.5 mM ${,}^{15}$N serine (609005, Lot: MBBB0411V; Sigma Aldrich) and ${,}^{15}$N glycine (299294, Lot: MBBC7772, CAS: 7299-33-4; Sigma Aldrich). Half of the labelled and non-labelled dishes were shifted to 4°C to induce cold acclimation for 5 days. The other half of the dishes remained in the control growth chamber with optimal temperature fluctuations of 25°C for 16 h and 18°C for 8 h.

### (c) Plant harvest and phenotyping

For phenotyping, each individual plant was considered a biological replicate. In total, 12 dishes were used; one per treatment and per time point. Six cold-germinated and six control plants were harvested each day from day zero until day five. Immediately, the seedlings were scanned to determine the length, width, volume and further root growth parameters. Afterwards, the roots of each plant were harvested separately, and the excess medium was completely removed with a paper towel. Samples were immediately weighed for FW, wrapped in a folded piece of Pergamin paper and dried for 70 h at approximately 70°C and weighed again for DW. For all subsequent analyses, root tips were collected in 1.5 cm segments on the fifth day of acclimation. Labelled and non-labelled plants reared at cold and control temperatures were collected for primary metabolome and complex-ome/ribo-proteomic assays. Additionally, root segments of plants from the remaining 60 dishes were harvested by pooling root segments from five dishes per biological replicate (for a total of three biological replicates per labelling—temperature combination). Briefly, 1.5 cm segments of the root tip were collected by using a sharp blade and flash-frozen with liquid nitrogen. By handling the plant material in frozen conditions, root pools were ground in a pre-frozen mortar and pestle, followed by preparation of 200 and 60 mg aliquots for complex-ome/ribo-proteomic and primary metabolome analyses, respectively. Ground plant material was stored at −80°C until further analysis.

### (d) Morphometric image processing

Images of the complete roots were acquired with an image analysis system and scanner (Perfection V800 Photo, Epson). Subsequently, software winRHIZO (Regent Instruments Inc., version released as 2019a) was used to delineate the root tissue and quantify the relevant morphological variables, including root length, root average diameter, root volume, number of forks or bifurcations, number of tips and root length per volume. The software parameters included ImgType - Grey, CalibMeth - Intr, TPU Units - cm, PxSizeH - 0.006353, PxSizeV - 0.006347 [CalFile] - Scanner. Cal, PxClassif - GreyThdAutom-57, Filters - Smooth Off Area Off LWRatio Off and Fractal PxMin PxMax - Off.

### (e) Primary metabolome analysis

To extract metabolites, 360 µl of pre-cooled extraction mix containing methanol:chloroform:water (2.5:1 (v/v)) and 30 µl of U-${,}^{13}$C sorbitol (0.2 mg ml^−1^), which was used as an internal standard, was added to 60 mg flash-frozen, ground root tip tissue, vortexed vigorously and incubated at 70°C for 15 min. Once the samples were cooled to room temperature, 200 µl of CHCl_3_ was added and incubated at 37°C for 5 min with shaking. Phase separation was induced by adding 400 µl H_2_O, vortexed and centrifuged at 20 800 rcf for 10 min. A 160 µl aliquot from the upper polar phase was transferred to fresh 2 ml microvials and dried via vacuum centrifugation for 18 h at room temperature. The dried samples were stored at −20°C until further use.

Primary metabolites were analysed via GC–MS of methoxyaminated and trimethylsilylated metabolite preparations [44]. Metabolite extraction and chemical derivatization were performed as previously described. A C${,}_{10}$, C${,}_{12}$, C${,}_{15}$, C${,}_{18}$, C${,}_{19}$, C${,}_{22}$, C${,}_{28}$, C${,}_{32}$ and C${,}_{36}$ n-alkane mixture was added to each sample for retention index calculation. Samples were processed by using a Factor Four Capillary Column VF-5ms with dimensions of 30 m length, 0.25 mm internal diameter and 0.25 mm film thickness (Variant Agilent) mounted to an Agilent 6890N gas chromatograph with split/splitless injector and electronic pressure control up to 150 psi (Agilent, Böblingen, Germany). Mass spectrometric data were acquired through a Pegasus III time-of-flight mass spectrometer (LECO Instrumente GmbH, Mönchengladbach, Germany) and in parallel by using the same samples at high mass resolution via a micrOTOF-Q II hybrid quadrupole time-of-flight mass spectrometer (Bruker Daltonics, Bremen, Germany) with a multipurpose APCI source. Detailed GC–electron spray ionization TOF–MS settings were used as previously reported [44].

Metabolites were annotated and identified by using mass spectral and retention index matching to data of authenticated reference compounds from the Golm Metabolome Database [132].

### (f) Ribosome-enriched proteomics

#### (i) Protease considerations

Lys-C over Trypsin: Amino acids enriched in RNA protein-binding domains include histidine, arginine and lysine, which are basic amino acids. The r-proteome is enriched in basic amino acids to be able to bind rRNA. Trypsin cleaves peptide sequences at the C-terminus of lysine and arginine residues; thus, it would digest r-proteins into smaller pieces compared to Lys-C, which cleaves peptide sequences only at the C-terminal side of lysine residues. Thus, Lys-C cuts sets of basic proteins (such as the r-proteins) into significantly longer pieces compared to trypsin [48].

#### (ii) Ribosomal protein purification and processing

Cell lysis was induced in ground plant tissue by using previously reported methods [14,133], with minor modifications. Briefly, aliquots were placed into liquid nitrogen-cooled mortars, and mass spectrometry-friendly ribosome extraction buffer (MS_f_-REB) was added at a buffer (V) to tissue (FW) ratio of two. The extract was then homogenized for 20 min while the mortars stayed on ice to prevent the temperature from increasing. Large particles were filtered through a pre-made, autoclaved, and tip-amputated 5 ml pipette tip containing a ©Miracloth clog inside of the tip, and the filtrate was aliquoted in 2 ml microcentrifuge tubes. Samples were centrifuged at 14 000*g* for 20 min (4°C) to pellet insoluble cell debris, and supernatants were transferred to violet QIAshredder mini spin columns (Qiagen, Australia) and centrifuged again for 1 min. The sample volume was adjusted to 4.5 ml to fill the ultracentrifuge tubes until they were 50% filled. Subsequently, extracts were carefully loaded into thick-walled polycarbonate tubes with three-piece caps (10.4 ml, polycarbonate bottle with cap assembly, 16 × 76 mm—6Pk, 355603, Beckman Coulter, USA) prefilled with 2.5 ml SC solution.

MS_f_-REB: 0.2 M Tris, pH 9.0, 0.2 M KCl, 0.025 M EGTA, pH 8.0, 0.035 M MgCl${,}_{2}$, 1% (w/v) octyl beta-D-glucopyranoside (98%; O8001, Sigma Aldrich, Australia), 0.18 mM cyclohexamide (Sigma Aldrich, Australia), 5 mM dithiothreitol (R0861, Thermo Fisher, Australia), 1 mM phenylmethylsulfonyl fluoride (36978, Thermo Fisher, Australia) and 1× protease inhibitor cocktail (cat. no. P9599, Sigma Aldrich, Australia).

SC: 0.4 M Tris, pH 9.0, 0.2 M KCl, 0.005 M EGTA, pH 8.0, 0.035 M MgCl${,}_{2}$ × 6 H_2_O, 60% sucrose (Molecular Biology Grade; 573113, Sigma Aldrich, Australia), 0.18 mM cyclohexamide (Sigma Aldrich, Australia), 5 mM dithiothreitol (R0861, Thermo Fisher, Australia), 1 mM phenylmethylsulfonyl fluoride (36978, Thermo Fisher, Australia) and 1× protease inhibitor cocktail (cat. no. P9599, Sigma Aldrich, Australia).

Loaded samples were centrifuged at 4°C and 330 000*g*/60 000 r.p.m. for 4.5 h by using a TY 70.1Ti rotor (Type 70.1 Ti Rotor; Beckman Coulter, USA) loaded into an Optima XE-100 Ultracentrifuge (Beckman Coulter, USA). After centrifugation, the supernatant was removed, including the SC, with care being given that the only solution in contact with the pellet was the SC. Tubes were completely dried by placing them upside down for several minutes, and dried pellets were stored at −80°C until further usage. Ribosome-enriched pellets were resuspended in 60 µl of freshly prepared GuHCl to dissociate r-proteins from rRNA, and trifluoroaceticacid (TFA) was added to a 1% final volume to induce precipitation of nucleic acids. The solution was then centrifuged in a microcentrifuge at 20 800*g* for 20 min, and the supernatant was recovered. Protein content was determined in samples using the bicinchoninic acid kit (Thermo Scientific, United States) assay. As a control *E. coli* ribosomes (P0763S, NEB, Australia) were used in 4 µl aliquots (approx. 2000 A260 units that are equivalent to 102 µg of ribosomes and 23 µg of r-protein) to undergo the full protocol [48], confirming the integrity of ribosomal complexes when passing through the SCs solution and subsequent r-protein dissociation.

Protein amounts were standardized to the minimum concentration, i.e. 13.7 µg in 50 µl 6 M GuHCl, 1% TFA. Proteins were reduced and alkylated by adding tris(2-carboxyethyl)phosphine (77720, Thermo Scientific, United States) and iodoacetamide (A3221, Sigma Aldrich, Australia) to 10 and 55 mM, respectively, after which they were shaken for 45 min at 37°C. The alkylation step was performed in the dark. Acetonenitrile was then added to 70%, and a 10 : 1 ratio of magnetic beads (Hydrophilic-Part no: 45152105050250, GE Healthcare plus Hydrophobic-Part no: 65152105050250, GE Healthcare, Australia) was added and mixed with the solution. Beads were prepared according to the manufacturer’s instructions to a concentration of 20 µg µl^−1^ stock. The solution was allowed to sit for 20 min with two pipette mixes, with one being performed every 10 min. Tubes were placed on a magnetic rack (DynaMag-2; 12321D, Life Technologies) and allowed to separate for 30 s. Afterwards, washes were performed while the tubes remained in the rack. One millilitre of neat acetonitrile was added for 10 s and removed, followed by 1 ml of 70% ethanol for 10 s, after which it was removed. Tubes were removed from the rack, and 1 : 10 protein (µg) to digestion buffer (µl) was immediately added. The digestion buffer (25 mM triethylammonium bicarbonate) contained the Lys-C protease (P8109S, NEB, Australia) at a 1:20 protease to protein ratio. Samples were incubated for 18 h at 37°C at 1000 r.p.m. in a thermomixer (Eppendorf, Australia). Subsequently, TFA was added to 1% to quench the reaction, after which the tubes were placed on the magnetic rack, and the supernatant was twice transferred to new tubes. Finally, a centrifugation step at 20 800*g* was performed to remove any residual beads, and only 90% of the supernatant was recovered. The recovered fraction was frozen for an hour at −80°C and then freeze-dried. Peptides were resuspended in MS-loading buffer (2% Acn + 0.05% TFA) and loaded into an LC–MS/MS platform.

#### (iii) Liquid chromatography-tandem mass spectrometry analysis

All of the samples were analysed via nano-electrospray ionization (ESI)-LC-MS/MS. The Nano-LC system, which is known as the Ultimate 3000 RSLC (Thermo Fisher Scientific, San Jose, CA, USA), was setup with an Acclaim Pepmap RSLC analytical column (C18, 100 Å, 75 µm × 50 cm; Thermo Fisher Scientific, San Jose, CA, USA) and Acclaim Pepmap nanotrap column (75 µm × 2 cm, C18, 100 Å) and controlled at 50°C. Solvent A included 0.1% v/v formic acid and 5% v/v dimethyl sulfoxide (DMSO) in water, and solvent B included 0.1% v/v formic acid and 5% DMSO in acetonitrile (ACN). The trap column was loaded with digested peptides at an isocratic flow of 3% ACN containing 0.05% TFA at 6 µl min^−1^ for 6 min, followed by the switch of the trap column in parallel to the analytical column.

To measure peptides from the barley experimental samples, the gradient settings for the LC runs (at a flow rate of 300 nl min^−1^) were set as follows: solvent B at 3–23% in 89 min, 23–40% in 10 min, 40–80% in 5 min, maintained at 80% for 5 min before dropping to 3% in 0.1 min and equilibration at 3% of solvent B for 9.9 min. An Exploris 480 Orbitrap mass spectrometer (Thermo Fisher Scientific, San Jose, CA, USA) with nano-ESI source at positive mode was employed to execute the MS experiments by using settings of spray voltages, ion funnel radio frequency (RF), and capillary temperature level at 1.9 kV, 40%, and 275°C, respectively. The MS data were acquired with a 3 s cycle time for one full scan MS spectrum and for as many data-dependent, higher-energy C-trap dissociation (HCD)–MS/MS spectra as possible. Full scan MS spectra feature ions at m/z of 300−1,600, a maximum ion trapping time of 25 msec, an auto gain control target value of 3 × 10^−6^, and a resolution of 1 20 000 at m/z 200. An m/z isolation window of 1.2, an auto gain control target value of 7.5 × 10^−4^, a 30% normalized collision energy, a first mass at m/z of 120, an automatic maximum ion trapping time, and a resolution of 15 000 at m/z 200 were used to perform data-dependent HCD–MS/MS of precursor ions (charge states from 2 to 6).

To measure peptides from commercially available *E. coli* ribosomes, gradient settings for the LC runs at a flow rate of 300 nl min^−1^ were as follows: solvent B at 3–23% in 59 min, 23–40% in 10 min, 40–80% in 5 min, maintained at 80% for 5 min before dropping to 3% in 0.1 min and equilibration at 3% of solvent B for 9.9 min. An Eclipse Orbitrap mass spectrometer (Thermo Fisher Scientific, San Jose, CA, USA) with a nano ESI source in positive mode was employed to perform the MS experiments by using settings of spray voltages, ion funnel RF and capillary temperature level at 1.9 kV, 30% and 275°C, respectively. The MS data were acquired with a 3 s cycle time for one full scan of MS spectra and for as many data-dependent, HCD–MS/MS spectra as possible. Full scan MS spectra feature ions at m/z 375−1500, a maximum ion trapping time of 50 ms, an auto gain control target value of 4 × 10^–5^ and a resolution of 120 000 at m/z 200. An m/z isolation window of 1.6, an auto gain control target value of 5 × 10^–^${,}^{4}$, a 30% normalized collision energy, a maximum ion trapping time of 22 ms and a resolution of 15 000 at m/z 200 were used to perform data-dependent HCD–MS/MS of precursor ions (charge states from 2 to 6).

Complete dataset proteomics submissions have been deposited to the ProteomeXchange Consortium [134] via the PRIDE [135] partner repository with the dataset identifiers PXD032923 for *H. vulgare* experimental samples (doi: 10.6019/PXD032923) and PXD032938 for *E. coli* control samples (doi: 10.6019/PXD032938).

### (g) Data analyses

#### (i) Phenotyping

RGRs were calculated using fresh, DW, and varied phenotype measurements by using R programming language with the electronic supplementary material, equation 8.1. The output units were mg × (mg${,}^{-1}$ × h${,}^{-1}$). W is the total weight accumulation at *t*_f_ and is used in the electronic supplementary material, equation 8.1 to transform the growth rates into fractions of the final weight. Delta *W* or *dW* is the change in fresh or DW accumulation in milligrams from time 0 or non-germinated until delta *t* or *dt*, which represents hours after germination. Thus, the assumption of an initial root weight of 0 leads to *dW${,}_{t}$* being represented by the weight measurement at time point *t*. The Tukey HSD test was performed after an ANOVA with a confidence level of 95%.

#### (ii) Homology alignments

Paralogues from r-proteins were identified by performing homology alignments with the Needleman–Wunsch global alignment algorithm exactly as we have previously done [14]. For the alignments, we used both Arabidopsis and barley as target and source sequences and used both alignments into the best paralogue identification (alignment details and scores are presented in the electronic supplementary material, table S4H).

#### (iii) Primary metabolome

Amino acid abundances were analytically derived from GC–EI–ToF–MS acquired data by using software TagFinder [136] and the Golm Metabolome Database [132]. Extraction, standardization, derivatization and GC–MS analytics were performed according to Erban *et al*. [44]. Three biological replicates were measured in technical triplicates. For highly abundant metabolites reaching the detection limit, measurements of all of the samples were repeated with a 1 : 30 dilution (split) of extracts. Compounds were manually annotated in TagFinder, and representative tags for each metabolite were chosen. Moreover, metabolome data were normalized to the levels of an internal ^13^C_6_ sorbitol standard (CAS 121067-66-1); in addition, the background levels of the blanks were subtracted, and data were normalized to the FW of plant material in each sample. For the electronic supplementary material, table S2, primary metabolome data were analysed with ‘OmicsUnivariateStats. R’ function of the RandoDiStats R package. Herein, missing values were replaced by a small normally distributed numeric vector. Additionally, the fold change in metabolite abundances under cold conditions, as well as the logical induction of metabolites (absence–presence scenarios), were calculated.

${,}^{15}$N enrichment percentages of labelled metabolite pools were analytically derived from a multiplexed GC–EI–ToF–MS and GC–APCI–qToF–MS platform. In the first case, the workflow entailed the baseline correction of the raw chromatogram files by using the vendor software and transformation into CDF files. Pre-processing of the chromatograms for increasing the quality of the data matrices (internal standard normalization and chromatogram alignment, mass scan width synchronization) was performed by using TagFinder. Similarly, in the latter case, the vendor software was used to identify the amino acid peaks in the chromatograms. Peaks were manually mined and integrated to derive relative abundances. Every step of the targeted manual annotation of N-containing mass tags is shown in the electronic supplementary material, table S3. Owing to the fact that ${,}^{15}$N feeding can cause differential abundances in monoisotopic fragments from the same amino acid analyte depending on the lack or presence of an N atom, multiple fragments per analyte and multiple isotopologues per fragment were considered. Thus, to account for the stable isotope variation, the correlation between fragment abundances was modified from a classical correlation among monoisotopic abundances to a correlation of the sum from all of the measured isotopologues for each fragment pair. Finally, for each amino acid analyte, three or more fragments were considered to provide well-rounded annotation. From the final list of fragments, the most abundant ones (i.e. in the linear range of MS detection) were selected to calculate the percentage of ${,}^{15}$N enrichment. Only fragments with null enrichment in the control were allowed to pass to the next stage. When all of the fragments presented residual ‘enrichment’ in non-labelled samples, this method considered these compounds plagued with technical bias; as such, the mean ‘enrichment’ in non-labelled samples was subtracted from the labelled samples, and those fragments in which the final variance in control ‘enrichment’ was minimal were further used for analysis. Furthermore, when multiple fragments satisfied the criteria to be useful as a proxy for amino acid synthesis, the most accurate fragments were defined to be those fragments with the lowest relative standard deviation across technical triplicates and biological replicates (electronic supplementary material, file S1). Subsequently, by using the molecular formula information per fragment, natural isotopic abundance (NIA) corrections and percentage of enrichment calculations [47] were performed, followed by a statistical comparison using the RandoDiStats R package (Tukey HSD test was performed after an ANOVA, with a confidence level of 95%).

#### (iv) Plant protein synthesis rates (*K*${,}_{s}$)

Two main approaches were used to derive our own calculations of protein synthesis rates, which are both detailed in the electronic supplementary material calculations, where all the steps taken to analyse the ribosome-enriched proteome purified in this study are also detailed (electronic supplementary material calculations, section ‘Ribosome Enriched Proteome Data Analyses’) [138] and [139].

## 5. Conclusion

With careful consideration of the ${,}^{15}$N isotope flux and plant phenotype, we were able to monitor tracer incorporation into digested peptides of proteins at the complex-ome proteome level and compare them between experimental conditions. Our strategy can be applied to any system that transitions between different biological steady states to study the dynamics of protein synthesis, as long as the right variables can be measured. We have made our equations and complete bioinformatics method available as a public R package, i.e. the ProtSynthesis R package. We applied this strategy to understand the transition of proliferative root tissue from germinating barley seedlings to a cold-acclimated state. The proliferating root tissue of germinating barley seedlings undergoing cold acclimation seems to require ribosome biogenesis to overcome the initial stimulus, as previously reported for Arabidopsis. In addition, plants build remodelled and heterogeneous ribosomes that directly or indirectly cause a shift in the proteome. To characterize the heterogeneity, we mapped the relative stoichiometry of ribosome-assembled r-proteins and their synthesis rates by using proteome-wide ${,}^{15}$N labelling to determine which part of the r-proteome shift is owing to synthesis and which part is owing to the reuse of pre-existing r-proteins. We can currently conclude that plants significantly and differentially modulate the relative synthesis rates of ribosome-bound r-proteins when confronted with environmental factors, such as a shift to suboptimal temperature; in addition, such modulation appears to be independent of *de novo* ribosome assembly. Moreover, ribosomes remodelled in the cold exhibit subtractive heterogeneity around the PET and an accumulation of specific r-proteins in both 40S and 60S subunits that are structurally linked to key inter-subunit bridges. In addition, we examined general proteome shifts and found that 43S and 48S translation initiation complexes are preferentially synthesized and accumulate during cold, thus leading to a higher requirement for 60S subunits, which are at a constant ratio with 40S subunits but appear to be insufficient to form elongation-competent 80S monosomes and solve the over-accumulation of initiation complexes during cold. Therefore, we hypothesize that 60S subunits are not able to bind all of the translation initiation complexes; consequently, they selectively associate with specific transcript-associated 48S complexes. This hypothesis is supported by the cold-induced heterogeneity, which mainly relates to the association of 40S and 60S subunits and as such could be a way to identify translational needs inherent to the cold context. The other major shift in the newly synthesized proteome is a response to protein aggregation and misfolding, which we propose is linked to missing r-proteins around the PET in cold-remodelled ribosomes. This mechanism may represent a second layer of translational control that allows ribosomes to misfold and target the part of the proteome that is currently not needed for degradation. From this study, we can currently conclude that there are major responses in the plant translational apparatus during cold that cause ribosomes to build a proteome to respond to the consequences of their own structural adaptations. Concomitantly, cold-heterogeneous ribosomes are able to directly or indirectly cause proteome shifts to remodel the cellular membrane and cell wall as part of the mechanism to transition to an acclimated state and eventually resume growth.

## Data Availability

The proteomic datasets that support the findings in this study are publicly available as full submissions deposited in the PRIDE repository under project accessions [140] and [141]. The ProtSynthesis R package can be installed using the instructions provided in the GitHub repository [142]. All electronic supplementary materials associated with this manuscript can be accessed via FigShare [143].
